# Desempenho do Eletrocardiograma no Diagnóstico da Hipertrofia Ventricular Esquerda em Hipertensos Idosos e Muito Idosos

**DOI:** 10.36660/abc.20200600

**Published:** 2021-07-27

**Authors:** Fernando Focaccia Povoa, Braulio Luna, Henrique Tria Bianco, Celso Amodeo, Rui Povoa, Maria Teresa Nogueira Bombig, Roberto Dischinger Miranda, Simone Matheus Fischer, Maria Cristina Oliveira Izar, Francisco A. H. Fonseca

**Affiliations:** 1 Universidade Federal de São Paulo Escola Paulista de Medicina São Paulo SP Brasil Universidade Federal de São Paulo - Escola Paulista de Medicina, São Paulo, SP - Brasil.; 2 Universidade Federal de São Paulo São Paulo SP Brasil Universidade Federal de São Paulo, São Paulo, SP - Brasil.

**Keywords:** Eletrocardiografia/métodos, Idoso, Hipertrofia Ventrículo Esquerdo, Hipertensão, Insuficiência Cardíaca, Acidente Vascular Cerebral, Infarto do Miocárdio

## Abstract

**Fundamento:**

A hipertrofia ventricular esquerda (HVE) é um importante fator de risco cardiovascular, independente da hipertensão arterial. Apesar da evolução dos exames de imagem, o eletrocardiograma (ECG) ainda é o mais utilizado na avaliação inicial, porém, com baixa sensibilidade.

**Objetivo:**

Avaliar o desempenho dos principais critérios eletrocardiográficos para HVE em indivíduos hipertensos idosos e muito idosos.

**Métodos:**

Em coorte de hipertensos foram realizados ECGs e EcoDopplercardiogramas (ECO), e separados em três grupos etários: <60 anos, Grupo I; 60-79 anos Grupo II; e ≥80 anos, Grupo III. Os critérios eletrocardiográficos mais utilizados foram aplicados para o diagnóstico da HVE: Perúgia; Peguero-Lo Presti; Gubner-Ungerleider; Narita; (Rm+Sm) x duração; Cornell voltagem; Cornell voltagem duração; Sokolow-Lyon voltagem; R de aVL ≥11 mm; RaVL duração. Na avaliação do desempenho desses critérios, além da sensibilidade (Sen) e especificidade (Esp), foram analisadas as “Odds Ratios diagnóstico” (DOR). Consideramos p-valor <0,05 para as análises, com testes bi-caudais.

**Resultados:**

Em 2.458 pacientes, a HVE estava presente pelo ECO em 781 (31,7%). Nos Grupos I e II, os melhores desempenhos foram para os critérios de Narita, Perúgia, (Rm+Sm) x duração, sem diferenças estatísticas entre eles. No Grupo III (muito idosos) os critérios de Perúgia e (Rm+Sm) x duração tiveram os melhores desempenhos: Perúgia [44,7/89,3; (Sen/Esp)] e (Rm+Sm) duração [39,4%/91,3%; (Sen/Esp), p<0,05)], com os melhores resultados de DOR:6,8. Isto sugere que nessa população de muito idosos esses critérios têm maior poder discriminatório para separar pacientes com HVE.

**Conclusão:**

Nos hipertensos muito idosos os critérios eletrocardiográficos de Perúgia e (Rm+Sm) x duração apresentaram os melhores desempenhos diagnósticos para HVE.

## Introdução

A hipertrofia ventricular esquerda (HVE) é um importante preditor de eventos cardiovasculares. Quando diagnosticada pelo eletrocardiograma (ECG), associa-se com aumento do risco de eventos como acidente vascular cerebral, infarto do miocárdio, insuficiência cardíaca, morte súbita e insuficiência vascular periférica. Tais desfechos independem, inclusive, da presença ou ausência de hipertensão arterial sistêmica (HAS). ^1^^-^^3^ Nesse cenário, o ECG é uma ferramenta de baixo custo econômico amplamente utilizada, apesar da baixa sensibilidade (Sen) diagnóstica para HVE. ^4^ Diversos critérios eletrocardiográficos para HVE já foram publicados, com diferentes Sens e especificidades (Esps). Todavia, poucos são utilizados na prática clínica. Isso decorre, em geral, da baixa precisão diagnóstica desses critérios quando aplicados em população com diferentes manifestações cardiovasculares e características epidemiológicas próprias, tais como idade, raça, antecedentes patológicos, etc. ^5^ Esses critérios apresentam boa Esp, mas baixa Sen. Além disso, a Sen apresenta amplas variações, dependendo da população e das doenças que desencadearam a hipertrofia ventricular. ^6^

Com o envelhecimento da população, cresce em importância o conhecimento sobre as doenças cardiovasculares, e a HAS desponta com maior prevalência. Nesse sentido, o ECG tem papel fundamental não apenas no diagnóstico, mas também na estratificação de risco de indivíduos idosos, propiciando identificar situações que ainda não tiveram expressão clínica. ^7^ Em pacientes idosos (≥ 60 e < 80 anos) e muito idosos (≥ 80 anos), são escassos os estudos em hipertensos em que o ECG foi testado em precisão diagnóstica para HVE. ^8^

Por conseguinte, o objetivo deste estudo é avaliar o desempenho dos principais critérios eletrocardiográficos no diagnóstico da HVE em amostra ambulatorial de pacientes hipertensos idosos e muito idosos.

## Métodos

**Figura 1:**
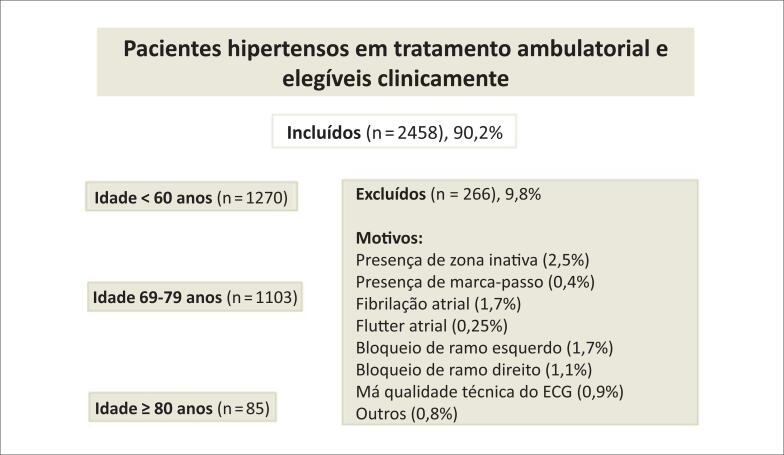
Fluxograma da coorte estudada. ECG: eletrocardiograma.

Foram analisados 2458 ECGs e ecocardiogramas (ECOs) de pacientes hipertensos em tratamento e acompanhamento no Setor de Cardiopatia Hipertensiva da Universidade Federal de São Paulo, no período entre 2006 a 2019. Todos os pacientes faziam uso regular de medicações anti-hipertensivas. Foram excluídos indivíduos com doença orovalvar, doença arterial coronariana aguda ou crônica, distúrbios de ritmo cardíaco, bloqueios de ramo do feixe de His, síndrome de pré-excitação, distúrbios eletrolíticos ou alteração eletrocardiográfica que pudessem interferir na análise, conforme fluxograma ( Figura 1 ). Os pacientes foram classificados em três faixas etárias: Grupo I, idade < 60 anos; Grupo II, idoso (60-79 anos) e Grupo III, muito idoso (≥ 80 anos). ^9^

O protocolo do estudo foi aprovado pelo Comitê de Ética em Pesquisa da Universidade Federal de São Paulo- Escola Paulista de Medicina (CAAE: 29732020.6.0000.5505).

### ECG

O ECG de 12 derivações em repouso foi realizado com o paciente em posição supina, na velocidade padrão de 25 mm/s e calibração padronizada para 1,0 mv/cm (aparelho Dixtal EP3^®^ e Cardiocare 2000 Bionet^®^). O traçado foi analisado com paquímetro devidamente calibrado e lupa de alta precisão, permitindo aumento de aproximadamente cinco vezes, para maior precisão da análise. Essas análises foram realizadas por um observador, cardiologista experiente, que desconhecia as características clínicas e epidemiológicas dos pacientes. Foram aferidas as seguintes variáveis: o eixo e duração dos complexos QRS, a distância entre as ondas R (intervalo R-R), intervalo QT, amplitudes da onda R em D_I_, aVL, V_5_ e V_6_, as amplitudes da onda S em V_1_, V_2_, V_3_ e V_4_, padrão *strain* em V_5_ e V_6_, além da maior amplitude das ondas R e S nas derivações do plano horizontal. Esses dados foram alocados em uma planilha de Excel^®^, dedicada para a respectiva análise.

A análise de reprodutibilidade das medidas e da aplicação dos critérios eletrocardiográficos foi realizada por dois cardiologistas do Setor de Cardiopatia Hipertensiva, que interpretaram de forma independente 100 traçados de ECG retirados aleatoriamente da amostra. Esses traçados foram selecionados por meio de lista gerada por *software* dedicado, em que os quatro primeiros dígitos eram associados ao registro dos pacientes no banco de dados.

### Descritores de HVE avaliados:

1. (Rmáx + Smáx) x duração do complexo QRS: somatória da maior amplitude da onda S com a maior amplitude da onda R no plano horizontal (em mm), multiplicando-se o total pela duração do complexo QRS (em segundos). Estabelece-se o diagnóstico de HVE se o resultado for ≥ 2,8 mm.s. ^10^
2. Critério de Sokolow-Lyon voltagem: SV_1_ + RV_5_ ou V_6_ ≥ 30 mm e ≥ 35 mm. ^11^
3. Critério de Cornell voltagem: RaVL + SV_3_ ≥ 20 mm para mulheres e ≥ 28 mm para homens. ^12^
4. Critério de Cornell duração: (RaVL + SV_3_).duração do QRS. Para mulheres adicionar 8 mm ≥ 2440 mm.ms. ^13^
5. Escore de pontos de Romhilt-Estes: amplitude de R ou S ≥ 30 mm no plano horizontal ou ≥ 20 mm no plano frontal, padrão *strain* em V_5_ ou V_6_ (se em uso de digital vale apenas um ponto) e crescimento do átrio esquerdo pelo índice de Morris – esses dados individualmente somam três pontos; eixo elétrico de ÂQRS acima de menos 30 graus soma dois pontos; duração de QRS ≥ 90 ms em V_5_ ou V_6_ ou tempo de ativação ventricular ≥ 50 ms em V_5_ ou V_6_ somam um ponto. Por esse escore, a HVE é diagnosticada quando a soma dos pontos é ≥ 5. ^14^
6. Onda R de aVL ≥ 11 mm. ^15^
7. Escore de Perúgia: HVE é diagnosticada pela presença de um ou mais dos seguintes achados: critério de Cornell, considerando o limite para mulheres ≥ 20 mm e para homens ≥ 24 mm, escore de Romhilt-Estes e padrão *strain* . ^16^
8. Critério de Peguero-Lo Presti: maior S em qualquer derivação + SV_4_. Se o resultado for ≥ 2,8 mV em homens e ≥ 2,3 mV em mulheres, estabelece-se o diagnóstico de HVE. ^17^
9. Critério de Narita: onda R de D_1_ + onda S de V_4_, se ≥ 1.6 mV em homem e ≥ 1.4 mV em mulheres. ^18^
10. Escore de Gubner-Ungerleider: RD_1_ +SV_3_ > 25 mm. ^19^
11. RaVL produto: RaVL x duração QRS ≥ 1030 mm.ms. ^20^
12. Relação V_6_/V_5_ > 1. ^21^

### Ecodopplercardiograma transtorácico

Todos os exames foram realizados no Setor de Ecodopplercardiografia do Hospital São Paulo/Unifesp com aparelhos ATL^®^ 1500, USA, de acordo com protocolos e diretrizes especializadas, sendo executados por profissionais qualificados e com mais de 15 anos de experiência. O paciente era posicionado em decúbito lateral esquerdo, com imagens obtidas a partir das janelas estudadas (paraesternal longitudinal eixo longo, paraesternal longitudinal eixo curto, quatro câmeras, duas câmeras e modo M) simultaneamente ao registro do ECG. As seguintes medidas foram estudadas, de acordo com as recomendações da Convenção de Penn: tamanho do ventrículo esquerdo (VE) em sístole e diástole, espessura do septo interventricular e da parede posterior do VE no final da diástole, volumes diastólico e sistólico finais. ^22^ A massa do VE foi indexada para a superfície corpórea para ajuste das diferenças do tamanho do coração, a depender de cada paciente.

A massa do VE para a avaliação da presença de HVE foi calculada pelo Eco-Dopplercardiograma, conforme as recomendações da *American Society of Echocardiography/European Association of Echocardiograph* , de 2015, considerando HVE quando o índice de massa do ventrículo esquerdo (IMVE) for ≥ 96 g/m^2^ para a população feminina e ≥ 116 g/m^2^ para a população masculina. ^23^

### Análise estatística

As variáveis contínuas foram expressas em média (DP). Variáveis categóricas apresentadas em percentagens. Para análise do desempenho dos critérios eletrocardiográficos na HVE, foram utilizadas as medidas de Sen e Esp com os respectivos intervalos de confiança de 95% (IC95%), além da razão de chance diagnóstioca ( *diagnostic odds ratio* , DOR), que expressa a eficácia global de uma medida sem sofrer influência da prevalência, como ocorre com o valor preditivo positivo e negativo. Construímos também as curvas *receiver operating characteristic* (ROC) para os três grupos (GI, GII, GIII), considerando os critérios eletrocardiográficos que tiveram os melhores desempenhos. A DOR também é uma medida de precisão, usada para estimar o poder discriminativo e comparar a precisão entre os testes. ^24^

A reprodutibilidade interobservador foi avaliada pelo método de Kappa. ^25^ Nesse teste, admite-se que valores acima de 0,75 sejam considerados excelentes; entre 0,40 e 0,75; como boa concordância; e abaixo de 0,40, como de pobre concordância. Para a verificação de significância estatística, foi aplicado o teste de McNemar. ^26^ Este teste foi aplicado para avaliar as diferenças estatística entre os resultados dos critérios eletrocardiográficos para a HVE em relação às Sens e Esps.Considerou-se estatisticamente significante quando o p obtido era <0,05. Todas as análises foram executadas em programa SPSS^®^ (versão 17.0, SPSS Inc., Chicago, IL, EUA).

## Resultados

Dos 2.458 participantes, 753 eram homens (30,6%) e 1.705 mulheres (69,4%). Desse total, 1.270 pacientes (51,6%) constituíram o Grupo I (<60 anos); 1103 (44,8%) o Grupo II (faixa etária entre 60 a 79 anos); e 85 (3,5%) o Grupo III (idade igual ou superior a 80 anos). A presença de HVE no ECO ocorreu em 345 (27,1%) no Grupo I; 398 (36,0%) no Grupo II (idosos) e em 38 (44,7%) no Grupo III (muito idosos), como mostrado na Tabela 1 .

**Tabela 1:** Características da população estudada conforme faixa etária, gênero, idade e presença ou ausência de HVE pelo ecocardiograma

Faixa etária		<60 anos	60-79 anos	≥80 anos
N total: (2458)		1270	1103	85
**Sexo, n (%)**				
	Feminino	908 (71,5%)	738 (66,9%)	59 (69,4%)
	Masculino	362 (28,5%)	365 (33,1%)	26 (30,5%)
Idade (anos), média (DP)		50,1 (7,4)	67 (5,2)	84 (3,9)
Peso (kg), média (DP)		74,4 (16,1)	70,5 (12,9)	64,8 (12,8)
Altura (m), média (DP)		1,61 (0,09)	1,60 (0,07)	1,59 (0,09)
IMC, média (DP)		28,58 (5,61)	27,53 (4,63)	25,65 (4,51)
SC (m^2^), média (DP)		1,75 (0,21)	1,70 (0,18)	1,63 (0,18)
**Cavidades do VE (cm), média (DP)**				
Septo IV		0,98 (0,17)	1,00 (0,17)	1,02 (0,15)
Parede posterior		0,95 (0,16)	0,96 (0,15)	0,96 (0,13)
Diâmetro diastólico		4,78 (0,52)	4,79 (0,57)	4,80 (0,65)
Sem HVE no ECO		924	705	47
HVE no ECO, n (%)		345 (27,1%)	398 (36,0%)	38 (44,7%)
IMVE (g/m^2^), média (DP)		93,03 (28,79)	98,33 (27,65)	102,70 (32,74)

IMC: índice de massa corpórea; SC: superfície corpórea; VE: ventrículo esquerdo; Septo IV: septo interventricular; HVE: hipertrofia ventricular wesquerda; ECO: Ecocardiograma; IMVE: índice de massa do ventrículo esquerdo. Nota: os dados são expressos como média (DP).

Na Tabela 2 encontram-se descritas a Sen e a Esp dos critérios eletrocardiográficos para HVE e os respectivos IC95%s. As DORs dos critérios avaliados estão descritos na Tabela 3 . Observou-se nos pacientes do Grupo I e II desempenhos similares para os critérios de Narita, Perúgia e (Rmáx+Smáx) x duração, que despontaram com os melhores resultados. Já o Grupo III, com pacientes muito idosos, tiveram melhor desempenho apenas os critérios de Perugia (Sen 44,7% e Esp de 89,3%) e (Rmáx+Smáx) x duração (Sen 39,4% e Esp 91,3%). As DORs desses critérios eletrocardiográficos também apresentaram maior resultado (DOR =6,8), demonstrando melhor eficácia para detectar ou excluir a HVE ( Tabela 3 ).

**Tabela 2:** Característica da população estudada conforme a faixa etária, gênero, idade e presença ou ausência de HVE pelo ecocardiograma

Critérios de HVE	GI (<60 anos)		GII (60-79 anos)		GIII (≥80 anos)	
	Sensibilidade	Especificidade	Sensibilidade	Especificidade	Sensibilidade	Especificidade
	(IC95%)	(IC95%)	(IC95%)	(IC95%)	(IC95%)	(IC95%)
**Perúgia**	32,2	91,7	35,6	88,5	44,7	89,3
	(27,3-37,1)	(89,8-93,3)	(31,1-40,5)	(85,9-90,6)	(30,1-60,2)	(77,4-95,3)
**(Rmáx+Smáx) x duração**	33,8	88,9	32,4	88,9	39,4	91,3
	(29,0-38,9)	(86,7-90,8)	(28,0-37,1)	(86,4-91,0)	(25,6-55,2)	(79,6-96,5)
**Peguero-Lo Presti**	20,2	96,6	17,8	96,7	34,2	89,3
	(16,3-24,7)	(95,2-97,6)	(14,3-21,9)	(95,1-97,8)	(21,2-50,1)	(77,4-95,3)
**Narita**	39,6	89,3	38,1	87,5	26,3	91,4
	(34,5-44,8)	(87,2-91,2)	(33,5-43,0)	(84,8-89,7)	(14,9-42,0)	(80,0-96,6)
**Romhilt-Estes**	16,1	96,4	14,5	95	21	93,6
	(12,6-20,4)	(95,0-97,4)	(11,4-18,3)	(93,1-96,4)	(11,0-36,3)	(82,8-97,8)
**Cornell voltagem: [≥28 mm (h); ≥20mm (m)]**	18,2	97,1	17,3	90,6	21	91,4
	(14,5-22,6)	(95,9-98,0)	(13,9-21,3)	(88,3-92,5)	(11,0-36,3)	(80,0-96,6)
**Sokolow-Lyon voltagem (≥30 mm)**	23,7	92,1	20,8	92,6	21	93,6
	(19,5-28,4)	(90,1-93,6)	(17,1-25,1)	(90,4-94,3)	(11,0-36,3)	(82,8-97,8)
**Sokolow-Lyon voltagem (≥35 mm)**	14,7	97,1	12	97,1	15,7	97,8
	(11,3-18,8)	(95,9-98,0)	(9,2-15,6)	(95,6-98,1)	(7,4-30,4)	(88,8-99,6)
**Cornell Voltagem duração (≥2440 mm.ms)**	20,5	96,1	20,1	95,3	21	91,4
	(16,6-25,0)	(94,6-97,1)	(16,4-24,3)	(93,5-96,6)	(11,0-36,3)	(80,0-96,6)
**Gubner-Ungerleider (≥25 mV)**	18,5	97,2	16	97	15,7	93,6
	(14,7-22,9)	(96,0-98,1)	(12,8-20,0)	(95,4-98,0)	(7,4-30,4)	(82,8-97,8)
**RaVL (≥11 mm)**	11,8	96,6	12,3	95,8	15,7	93,6
	(8,8-15,6)	(95,2-97,6)	(9,4-15,9)	(94,1-97,1)	(7,4-30,4)	(82,8-97,8)
**V_6_/V_5_ (>1)**	15,3	88,1	14	90	13,1	87,2
	(11,9-19,4)	(86,9-90,1)	(11,0-17,8)	(87,6-92,0)	(5,7-27,3)	(74,8-94,0)
**RaVL x duração**	8,9	98,2	11,8	97,5	7,8	97,8
	(6,3-12,4)	(97,2-98,9)	(9,0-15,3)	(96,1-98,4)	(2,7-20,8)	(88,8-99,6)

HVE: hipertrofia ventricular esquerda. Nota: Os valores de sensibilidade e especificidade são expressos com seus respectivos intervalos de confiança de 95% (IC95%), com a utilização do método estatístico de McNemar.

**Tabela 3:** DOR dos critérios eletrocardiográficos descritores de HVE conforme a faixa etária

Critérios de HVE	GI (<60 anos)	GII (60-79 anos)	GIII (≥80 anos)
**Perugia**	5,2 (3,8-7,2)	4,2 (3,1-5,8)	6,8 (2,2-20,9)
**(Rmáx + Smáx) produto** ≥2,8 mm.s	4,1 (3,0-5,5)	3,8 (2,8-5,2)	6,8 (2,0-23,0)
**Peguero-Lo Presti**	7,3 (4,6-11,3)	6,4 (3,9-10,4)	4,3 (1,3-13,7)
**Narita**	5,5 (4,0-7,4)	4,3 (3,2-5,8)	3,8 (1,09-13,4)
**Romhilt-Estes**	5,2 (3,3-8,1)	3,2 (2,1-5,0)	3,9 (0,9-15,9)
**Cornell Voltagem:** ≥28 mm (h);≥20 mm (m)	7,6 (4,7-12,3)	2,0 (1,4-2,8)	2,8 (0,7-10,3)
**Sokolow-Lyon voltagem** ≥30 mm	3,6 (2,5-5,1)	3,3 (2,2-4,7)	3,9 (0,95-15,9)
**Sokolow-Lyon voltagem** ≥35 mm	5,9 (3,6-9,7)	4,6 (2,7-8,0)	8,6 (0,99-75,12)
**Cornell Voltagem duração** ≥2440 mm.ms	6,3 (4,1-9,7)	5,1 (3,3-7,8)	2,8 (0,7-10,3)
**Gubner-Ungerleider** ≥25 mV	8,1 (5,0-13,2)	6,2 (3,7-10,3)	2,7 (0,6-11,8)
**RaVL** ≥11 mm	3,8 (2,3-6,2)	3,2 (2,0-5,2)	1,9 (0,3-12,1)
**V_6_/V_5_** >1	1,3 (0,9-1,9)	1,4 (1,0-2,1)	1,03 (0,29-3,6)
**RaVL.dur QRS** >103 mm.ms	5,5 (3,0-10,3)	5,4 (3,0-9,5)	3,9 (0,39-39,5)

Nota: Dados expressos como a DOR e respectivo intervalo de confiança de 95% (IC95%). HVE: hipertrofia ventricular esquerda; DOR: razão de chance diagnóstica (diagnostic odds ratio).

**Figura 2:**
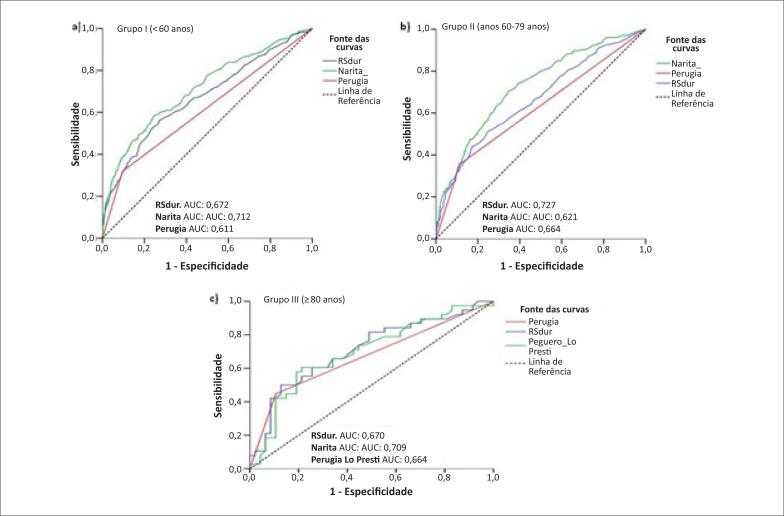
Curvas ROC dos grupos estudados. Grupo I (idade <60 anos); b) Grupo II (idade 60 a 79 anos); c) Grupo III (idade ≥80 anos). ROC: Receiver Operating Characteristic Curves; AUC: área sob a curva.

Na avaliação da reprodutibilidade na análise eletrocardiográfica, o nível de concordância entre os dois observadores foi de 0,82 e 0,94 (índice de kappa), considerados excelentes. O primeiro valor corresponde à duração do complexo QRS e o último à concordância dos critérios eletrocardiográficos. Foram construídas curvas ROC (Receiver Operating Characteristic), para os três grupos estudados ( Figura 2 ).

## Discussão

A HVE é um importante fator de risco cardiovascular independente de outras manifestações ou comorbidades. ^25^ Consequentemente, sua detecção por métodos diagnósticos de baixo custo e fácil acesso é extremamente relevante. Em pacientes hipertensos, a HVE é uma das manifestações pré-clínicas mais frequentes de lesão de órgão alvo cuja identificação leva à mudança na estratificação do risco e maior agressividade no tratamento. ^27^ Por sua vez, o ECG é um exame de baixo custo que, embora apresente baixa Sen, tem alta Esp e reprodutibilidade, e por isso é amplamente utilizado. Sofre, entretanto, influência de diversos fatores, como obesidade, tabagismo, gênero e, principalmente, idade. ^28^

A melhor avaliação da massa ventricular é feita pela ressonância nuclear magnética; porém, o custo econômico torna impraticável o emprego rotineiro na avaliação de pacientes hipertensos. ^29^ Nesse sentido, o ECO é utilizado como padrão ouro na avaliação da massa ventricular esquerda com alto grau de correlação e excelente reprodutibilidade intra e inter observador. No presente estudo, a referência para o diagnóstico de HVE foi o ECO-transtorácico. Foi aplicada a fórmula de Devereux-modificada para o cálculo da massa do VE, que apresenta boa correlação com a massa real do coração (r = 0,90; p < 0,001). ^30^

A população de idosos e muito idosos cresce cada vez mais no mundo. Já foi reconhecido que o controle dos fatores de risco, altamente prevalente nessa faixa etária, aumenta a expectativa de vida dessa população. ^31^ Por outro lado, sabe-se que a idade é um dos fatores que interfere na Sen do ECG na detecção da HVE. ^32^ Com o objetivo de identificar os melhores critérios eletrocardiográficos para o diagnóstico a presença de HVE em indivíduos idosos, cenário cada vez mais frequente nos consultórios e ambulatórios, avaliamos os principais índices eletrocardiográficos descritos na literatura e que foram utilizados em estudos epidemiológicos.

Em nossa coorte, o critério de Perúgia foi o que teve a maior Sen (44,7 %) nos pacientes muito idosos e idosos (35,6%), sem perda significante da Esp. Esse critério foi descrito por Schillaci et al., ^16^ em 1994 e faz o diagnóstico de HVE em pacientes hipertensos que apresentam no ECG pelo menos um entre os três parâmetros seguintes: padrão *strain* ; critério de Cornell voltagem modificado: SV_3_ + RaVL > 2,4mV no homem e 2,0 mV na mulher; ou escore de Romhilt-Estes ≥ 5. Os autores relataram Sen de 34% e Esp de 93%, havendo uma melhora razoável na Sen individual dos três critérios e sem redução da Esp. Embora abordem o desempenho do critério proposto em relação ao gênero e grau da massa do VE, não fazem alusão à influência da idade. Em nosso estudo, os pacientes com menos de 60 anos (Grupo I) apresentaram Sen e 32,2% e Esp de 91,7%, percentuais semelhantes aos relatados por Schillaci et al., ^16^ e houve um aumento progressivo da Se nos idosos (Grupo II) e muito idosos (Grupo III).

O critério que considerou a somatória da maior amplitude da onda R com a maior onda S multiplicada pela duração do QRS [(Rmáx + Smáx) x duração] também apresentou boa Sen na população de muito idosos (39,4%) com Esp de 91,3%. Na publicação original, não houve distinção de idade, e a Sen e a Esp foram de 35,2% e 88,7%, respectivamente. ^10^ Este critério, apesar de simples, teve resultado equivalente ao critério de Perúgia, pois não houve diferença estatística significante entre eles.

Recentemente um novo critério eletrocardiográfico para HVE foi proposto. Denominado como critério de Peguero-Lo Presti apresentava Sen de 62% e Esp de 90%. ^17^ Em nosso estudo, com pacientes muito idosos, (Grupo III) encontramos Sen de 34,2% e Esp de 89,3%. Já nos pacientes idosos (Grupo II), a Sen e Esp foram de 17,8% e 96,7%, respectivamente. Por fim, em 1270 pacientes com menos de 60 anos (Grupo I) a Sen foi de 20,2% e a Esp de 96,6%, resultados distintos dos relatados. Consideramos que as duas casuísticas são diferentes; os pacientes da amostra de Peguero- Lo Presti eram formados com paciente mais graves e alta prevalência de HVE (60%). Evidentemente que, em uma população com indivíduos mais graves, os testes diagnósticos tendem a ter maior Sen.

Na nossa casuística, a porcentagem de HVE nos grupos muito idoso, idoso e jovem foram respectivamente 44,7%, 36,0% e 27,1%. O critério de Narita, que considera a somatória da R em D_I_ com a amplitude da onda S em V_4_, apresentou boa Sen nos jovens e idosos (39,6% e 38,1%), respectivamente; entretanto, nos muito idosos a Sen foi de apenas 26,3%. Os critérios de Romhilt-Estes, Cornell voltagem e duração e Sokolow-Lyon ≥ 35 mm, apresentaram Sen muito semelhante nas três faixas de idade estudadas, com valores relativamente baixos que variaram entre 16,1 e 21%. Embora recomendados por diversas diretrizes de hipertensão arterial, esses critérios tiveram desempenho inferior. ^26^^,^^33^ Os demais critérios avaliados em nossa coorte não apresentaram resultados satisfatórios em relação à Sen, que variou de 8,9 a 18,5%.

Os índices eletrocardiográficos que tiveram melhor desempenho levaram em conta a amplitude da onda S em V_3_ ou V_4_ ou maior onda S. Provavelmente isso ocorre pelo fato de a HVE gerar maior projeção vetorial do complexo QRS no plano horizontal de orientação posterior. Na HVE a cavidade cresce posteriormente e para a esquerda, mudando a direção e a magnitude do vetor principal da despolarização. Desta forma nas precordiais V_3_ e V_4_ haverá um incremento da amplitude da onda S.

Constatamos que a maioria dos critérios eletrocardiográficos utilizados no diagnóstico da HVE perde Se com o aumento da idade da amostra. Isso, todavia, não ocorreu em relação ao critério de Perúgia e (Rmáx+Smáx) x duração, principalmente no Grupo III. Quando analisamos a DOR, que avalia a eficácia de uma mensuração independente da influência da prevalência e permite estimar a eficácia global do parâmetro, observamos que o critério de Perúgia e (Rmáx+Smáx) x duração apresentaram os maiores valores: DOR = 6,8. Dessa forma, nos Grupos I e II, a melhor Sen (39,6 e 38,1%) foi observada para o critério de Narita, que também apresentou alta Esp (89,3% e 87,5%). Todavia, para os pacientes muito idosos (Grupo III), os melhores desempenhos para o diagnóstico da HVE ocorreram com os critérios de Perúgia, e (Rmáx+Smáx) x duração, com Sen de 44,7% e 39,4%; e Esp de 89,3% e 91,3%, respectivamente. O critério de Sokolow-Lyon, amplamente utilizado em diversos estudos, e talvez o mais conhecido pelo médico devido à simplicidade de análise, mostrou baixa Se em todas as faixas etárias.

Nosso estudo mostrou que na idade avançada existe perda de desempenho de vários critérios para diagnóstico de HVE, justamente para esta população de alto risco cardiovascular. Assim, a principal contribuição de nossas observações foi o de detectar dois critérios eletrocardiográficos que se revelaram superiores na detecção da HVE em hipertensos muito idosos. Ainda, em muitas regiões e locais de atendimento não há a pronta disponibilidade para a realização de métodos diagnósticos por imagem, como o ECO. Dessa forma, o ECG no diagnóstico da HVE, utilizando-se dos critérios com melhor desempenho, pode ser ferramenta útil, de fácil acesso, não oneroso, de prática interpretação, e aplicável, sobretudo nas faixas etárias dos mais idosos.

### Limitações do estudo

A exclusão de doença arterial coronária neste estudo foi realizada pela história, por exames de imagem específicos, ou pela presença de ondas q patológicas no eletrocardiograma. Menor número de pacientes no grupo muito idosos em comparação com os jovens.

## Conclusões

Os resultados obtidos neste estudo sugerem que, em hipertensos muito idosos, os critérios eletrocardiográficos de Perúgia e [(Rmáx+Smáx) x duração] apresentaram os melhores desempenhos diagnósticos para a presença de HVE.
