# Supplementary material for: Desempenho do Eletrocardiograma no Diagnóstico da Hipertrofia Ventricular Esquerda em Hipertensos Idosos e Muito Idosos
Source: Arq Bras Cardiol. 2021 Jul 27;117(5):924–31. [Article in Portuguese] doi: 10.36660/abc.20200600 (PMC8682087; doi:10.36660/abc.20200600)
Supplement: Translation

# Translation: Performance of the Electrocardiogram in the Diagnosis of Left Ventricular Hypertrophy in Older and Very Older Hypertensive Patients

**Authors:** Fernando Focaccia Povoa, Braulio Luna, Henrique Tria Bianco, Celso Amodeo, Rui Povoa, Maria Teresa Nogueira Bombig, Roberto Dischinger Miranda, Simone Matheus Fischer, Maria Cristina Oliveira Izar, Francisco A. H. Fonseca

**Affiliations:** 1 São Paulo SP Brazil Universidade Federal de São Paulo - Escola Paulista de Medicina, São Paulo, SP - Brazil.; 2 São Paulo SP Brazil Universidade Federal de São Paulo, São Paulo, SP - Brazil.

**Keywords:** Electrocardiography/methods, Aged, Hypertrophy, Left Ventricular, Hypertension, Heart Failure, Stroke, Myocardial Infarction

## Abstract

**Background:**

Left ventricular hypertrophy (LVH) is an important cardiovascular risk factor, regardless of arterial hypertension. Despite the evolution of imaging tests, the electrocardiogram (ECG) is still the most used in the initial evaluation, however, with low sensitivity.

**Objective:**

To evaluate the performance of the main electrocardiographic criteria for LVH in elderly and very elderly hypertensive individuals.

**Methods:**

In a cohort of hypertensive patients, ECGs and doppler echocardiographies (ECHO) were performed and separated into three age groups: <60 years, Group I; 60-79 years Group II; and ≥80 years, Group III. The most used electrocardiographic criteria were applied for the diagnosis of LVH: Perugia; Pegaro-Lo Presti; Gubner-Ungerleider; Narita; (Rm+Sm) x duration; Cornell voltage; Cornell voltage duration; Sokolow-Lyon voltage; R of aVL ≥11 mm; RaVL duration. In evaluating the performance of these criteria, in addition to sensitivity (Sen) and specificity (Esp), the “Diagnostic Odds Ratios” (DOR) were analyzed. We considered p-value <0.05 for the analyses, with two-tailed tests.

**Results:**

In 2,458 patients, LVH was present by ECHO in 781 (31.7%). In Groups I and II, the best performances were for the criteria of Narita, Perugia, (Rm+Sm) x duration, with no statistical differences between them. In Group III (very elderly) the Perugia criteria and (Rm+Sm) x duration had the best performances: Perugia [44,7/89.3; (Sen/Esp)] and (Rm+Sm) duration [39.4%/91.3%; (Sen/Esp), p<0.05)], with the best PAIN results:6.8. This suggests that in this very elderly population, these criteria have greater discriminatory power to separate patients with LVH.

**Conclusion:**

In very elderly hypertensive patients, the Perugia electrocardiographic criteria and (Rm+Sm) x duration showed the best diagnostic performance for LVH.

## Introduction

Left ventricular hypertrophy (LVH) is an important predictor of cardiovascular events. When diagnosed by electrocardiogram (ECG), it is associated with increased risk of events such as stroke, myocardial infarction, heart failure, sudden death, and peripheral vascular disease. Indeed, these outcomes are independent of the presence or absence of systemic arterial hypertension (SAH). ^1^^–^^3^ In this scenario, ECG is a widely used low-cost tool, despite its low diagnostic sensitivity (Se) for LVH. ^4^ Several ECG criteria for LVH have already been published, with different Ses and specificities (Sps). However, few are used in the clinical practice. This usually results from the low diagnostic accuracy of these criteria when applied in a population with different cardiovascular manifestations and with specific epidemiological characteristics, such as age, race, clinical history, etc. ^5^ These criteria have good Sp, but low Se. Furthermore, Se varies greatly, depending on the population and on the diseases that led to ventricular hypertrophy. ^6^

With population aging, it has become increasingly important to improve understanding on cardiovascular diseases, and SAH stands out with the highest prevalence. In this sense, ECG has an essential role not only on the diagnosis but also on the risk stratification of older individuals, enabling to identify situations that had no clinical expression yet. ^7^ Few studies in older (≥ 60 and < 80 years) and very older (≥ 80 years) patients with hypertension investigated the diagnostic accuracy of ECG for LVH. ^8^

Therefore, the aim of this study is to assess the performance of the main ECG criteria in the diagnosis of LVH in an outpatient sample of older and very older hypertensive patients.

## Methods

The present study assessed 2458 ECGs and echocardiographies (ECHOs) of hypertensive patients under treatment and follow-up at the Department of Hypertensive Heart Disease of Universidade Federal de São Paulo from 2006 to 2019. All patients made regular use of antihypertensive drugs. Individuals with the following conditions were excluded: orovalvar disease, acute or chronic coronary artery disease, cardiac rhythm disorders, His bundle branch blocks, pre-excitation syndrome, electrolytic disorders, or ECG changes that could interfere with the analysis, as shown in the flowchart ( Fig.1 ). Patients were classified into three age groups: Group I, age < 60 years; Group II, older adults (60-79 years); and Group III, very older adults (≥ 80 years). ^9^

**Figure 1:** Flowchart of the study cohort. ECG: electrocardiogram.

The study protocol was approved by the Research Ethics Committee of Universidade Federal de São Paulo-Escola Paulista de Medicina (CAAE: 29732020.6.0000.5505).

### ECG

The 12-lead resting ECG was performed with the patient in the supine position, at a standard velocity of 25 mm/s, and in equipment calibrated for 1.0 mv/cm (Dixtal EP3^®^and Cardiocare 2000 Bionet^®^). ECG tracings were analyzed using a duly calibrated caliper and a high-precision magnifying glass, allowing for a nearly five-fold magnification in order to achieve a more accurate analysis. These analyses were conducted by an experienced cardiologist who had no knowledge of patients’ clinical and epidemiological characteristics. The following variables were measured: axis, QRS duration, the distance between R waves (R-R interval), QT interval, the amplitude of R waves in leads D_1_, aVL, V_5_and V_6_, the amplitudes of the S wave in V_1_, V_2_, V_3_and V_4_, and the strain pattern in V_5_and V_6_, as well as the largest amplitude of R and S waves in the horizontal plane leads. These data were entered into an Excel^®^spreadsheet specifically designed for the analysis.

The analysis of reproducibility of measures and application of ECG criteria was conducted by two cardiologists working at the Department of Hypertensive Heart Disease, who independently interpreted 100 ECG tracings randomly selected from the sample. These tracings were selected from a list generated by a dedicated software, in which the first four numbers were associated with patients’ records on the database.

### LVH descriptors assessed:

1. (Rmax + Smax) x QRS duration: sum of the greatest amplitude of S wave and the greatest amplitude of R wave in the horizontal plane (em mm), multiplied by QRS duration (in seconds). The diagnosis of LVH is established if the result is ≥ 2.8 mm.s. ^10^
2. Sokolow-Lyon voltage criteria: SV_1_+ RV_5_or V_6_≥ 30 mm and ≥ 35 mm. ^11^
3. Cornell's voltage criteria: RaVL + SV_3_≥ 20 mm for women and ≥ 28 mm for men. ^12^
4. Cornell's duration criteria: (RaVL + SV_3_) x QRS duration. For women add 8 mm, ≥ 2440 mm.ms. ^13^
5. Romhilt-Estes point score system: amplitude of R or S ≥ 30 mm on the horizontal plane or ≥ 20 mm on the frontal plane, strain pattern in V_5_or V_6_(when using digital, the score is only one point) and left atrial growth by the Morris index – these data individually add three points; electrical axis (ÂQRS) above less than 30 degrees adds two points; QRS duration ≥ 90 ms in V_5_or V_6_or ventricular activation time ≥ 50 ms in V_5_or V_6_add one point. Using this score, LVH is diagnosed when the sum of the points is ≥ 5. ^14^
6. R wave in aVL ≥ 11 mm. ^15^
7. Perugia score: HVE is diagnosed by the presence of one or more of the following findings: Cornell's criteria, considering the values ≥ 20 mm and ≥ 24 mm as limits for women and men, respectively; Romhilt-Estes score; presence of strain pattern. ^16^
8. Peguero-Lo Presti criterion: deepest S wave in any lead + SV_4_. The diagnosis of LVH is established if the result is ≥ 2.8 mV for men and ≥ 2.3 mV for women. ^17^
9. Narita criterion: R wave in limb lead 1 D_1_+ S wave in V_4_, if ≥ 1.6 mV in men and ≥ 1.4 mV in women. ^18^
10. Gubner-Ungerleider score: RD_1_+SV_3_> 25 mm. ^19^
11. RaVL product: RaVL x QRS duration ≥ 1030 mm.ms. ^20^
12. V_6_/V_5_ratio > 1. ^21^

### Transthoracic Doppler ECHO

The tests were performed in the Service of Doppler Echocardiography of Hospital São Paulo/Unifesp with the device ATL^®^1500, USA, according to specialized protocols and guidelines, by skilled professionals with more than 15 years of experience. The patient was placed on the left lateral decubitus position and the images were obtained from the assessed views (paraesternal long axis, paraesternal short axis, four-chamber, two-chamber, and M-mode views) simultaneously with the recording of the ECG. According to the recommendations of the Penn Convention, the following measurements were obtained: left ventricle (LV) size in systole and diastole; interventricular septum in diastole; end diastolic left ventricular posterior wall thickness; end systolic and diastolic volumes. ^22^ LV mass was indexed for body surface area to adjust differences in heart size, depending on each patient.

LV mass for the diagnosis of HVE was calculated by Doppler ECHO, according to 2015 recommendations of the American Society of Echocardiography/European Association of Echocardiograph, considering LVH when the left ventricular mass index (LVMI) is ≥ 96 g/m^2^for women and ≥ 116 g/m^2^for men. ^23^

### Statistical analysis

Continuous variables were expressed in mean (SD). Categorical variables were presented in percentages. For the analysis of the performance of the ECG criteria in LVH, measures of Se and Sp with their respective 95% confidence intervals (95%CIs), in addition to diagnostic odds ratio (DOR), which expresses the overall efficacy of a measure without the influence of prevalence, as it occurs with positive and negative predictive value. We also built receiver operating characteristic (ROC) curves for the three groups (GI, GII, GIII), considering the ECG criteria that had the best performances. DOR was also a measure of accuracy, used to estimate the discriminative power and to compare the accuracy between the tests. ^24^

Interobserver reproducibility was assessed by the kappa method. ^25^ In this test, values above 0.75 are defined as excellent agreement; from 0.40 to 0.75, as good agreement; and bellow 0.40, as poor agreement. Statistical significance was investigated using the McNemar's test. ^26^ This test was applied to assess the statistical differences between the results for ECG criteria for LVH with regard to Se and Sp. The level of significance was set at p < 0.05. All analyses were executed with the software SPSS^®^(version 17.0, SPSS Inc., Chicago, IL, USA).

## Results

Of the 2458 participants, 753 were men (30.6%) and 1705 were women (69.4%). Of this total, 1270 (51.6%) were included in Group I (<60 years); 1103 (44.8%) in Group II (from 60 to 79 years); and 85 (3.5%) in Group III (age 80 years or older). The presence of LVH in ECHO occurred in 345 (27.1%) in Group I; 398 (36.0%) in Group II (older adults), and in 38 (44.7%) in Group III (very older adults), as shown in Table 1 .

**Table 1:** Characteristics of the sample according to age group, gender, age, and presence or absence of LVH on echocardiography

Age group		<60 years	60-79 years	≥80 years
N total: (2458)		1270	1103	85
**Sex, n (%)**				
	Male	362 (28.5%)	365 (33.1%)	26 (30.5%)
	Female	908 (71.5%)	738 (66.9%)	59 (69.4%)
Age (years), mean (SD)		50.1 (7.4)	67 (5.2)	84 (3.9)
Weight (kg), mean (SD)		74.4 (16.1)	70.5 (12.9)	64.8 (12.8)
Height (m), mean (SD)		1.61 (0.09)	1.60 (0.07)	1.59 (0.09)
BMI, mean (SD)		28.58 (5.61)	27.53 (4.63)	25.65 (4.51)
BS (m^2^), mean (SD)		1.75 (0.21)	1.70 (0.18)	1.63 (0.18)
**LF cavities (cm), mean (SD)**				
IV septum		0.98 (0.17)	1.00 (0.17)	1.02 (0.15)
Posterior wall		0.95 (0.16)	0.96 (0.15)	0.96 (0.13)
Diastolic diameter		4.78 (0.52)	4.79 (0.57)	4.80 (0.65)
No LVH on ECO		924	705	47
LVH on ECHO, n (%)		345 (27.1%)	398 (36.0%)	38 (44.7%)
LVMI (g/m^2^), mean (SD)		93.03 (28.79)	98.33 (27.65)	102.70 (32.74)

BMI: body mass Index; BS: body surface; LV: left ventricle; IV Septum: interventricular septum; LVH: left ventricular hypertrophy; ECHO: echocardiography; LVMI: left ventricle mass index. Note: data are expressed as mean (SD).

Table 2 describes Se and Sp for the ECG criteria for LVH and their respective 95%CIs. DOR for the assessed criteria are described in Table 3 . Patients in Group I and II were found to have similar performances for the Narita, Perugia and (Rmax + Smax) x duration criteria, which showed the best results. Conversely, Group III, with very older patients, had a better performance only in the Perugia (Se 44.7% and Sp 89.3%) and (Rmax + Smax) x duration (Se 39.4% and Sp 91.3%) criteria. The DOR of these ECG criteria also showed better results (DOR = 6.8), showing better efficacy to detect or rule out LVH ( Table 3 ).

**Table 2:** Characteristics of the study population according to age group, gender, age, and presence or absence of LVH on echocardiography

Criteria for LVH	GI (< 60 years)		GII (60-79 years)		GIII (≥ 80 years)	
	Sensitivity	Specificity	Sensitivity	Specificity	Sensitivity	Specificity
	(95%CI)	(95%CI)	(95%CI)	(95%CI)	(95%CI)	(95%CI)
**Perugia**	32.2	91.7	35.6	88.5	44.7	89.3
	(27.3-37.1)	(89.8-93.3)	(31.1-40.5)	(85.9-90.6)	(30.1-60.2)	(77.4-95.3)
**(Rmax + Smax) x duration**	33.8	88.9	32.4	88.9	39.4	91.3
	(29.0-38.9)	(86.7-90.8)	(28.0-37.1)	(86.4-91.0)	(25.6-55.2)	(79.6-96.5)
**Peguero-Lo Presti**	20.2	96.6	17.8	96.7	34.2	89.3
	(16.3-24.7)	(95.2-97.6)	(14.3-21.9)	(95.1-97.8)	(21.2-50.1)	(77.4-95.3)
**Narita**	39.6	89.3	38.1	87.5	26.3	91.4
	(34.5-44.8)	(87.2-91.2)	(33.5-43.0)	(84.8-89.7)	(14.9-42.0)	(80.0-96.6)
**Romhilt-Estes**	16.1	96.4	14.5	95	21	93.6
	(12.6-20.4)	(95.0-97.4)	(11.4-18.3)	(93.1-96.4)	(11.0-36.3)	(82.8-97.8)
**Cornell's voltage [≥ 28 mm (m); ≥ 20mm (f)]**	18.2	97.1	17.3	90.6	21	91.4
	(14.5-22.6)	(95.9-98.0)	(13.9-21.3)	(88.3-92.5)	(11.0-36.3)	(80.0-96.6)
**Sokolow-Lyon voltage (≥ 30 mm)**	23.7	92.1	20.8	92.6	21	93.6
	(19.5-28.4)	(90.1-93.6)	(17.1-25.1)	(90.4-94.3)	(11.0-36.3)	(82.8-97.8)
**Sokolow-Lyon voltage (≥ 35 mm)**	14.7	97.1	12	97.1	15.7	97.8
	(11.3-18.8)	(95.9-98.0)	(9.2-15.6)	(95.6-98.1)	(7.4-30.4)	(88.8-99.6)
**Cornell's voltage duration (≥ 2440 mm.ms)**	20.5	96.1	20.1	95.3	21	91.4
	(16.6-25.0)	(94.6-97.1)	(16.4-24.3)	(93.5-96.6)	(11.0-36.3)	(80.0-96.6)
**Gubner-Ungerleider (≥ 25 mV)**	18.5	97.2	16	97	15.7	93.6
	(14.7-22.9)	(96.0-98.1)	(12.8-20.0)	(95.4-98.0)	(7.4-30.4)	(82.8-97.8)
**RaVL (≥ 11 mm)**	11.8	96.6	12.3	95.8	15.7	93.6
	(8.8-15.6)	(95.2-97.6)	(9.4-15.9)	(94.1-97.1)	(7.4-30.4)	(82.8-97.8)
**V_6_/V_5_(> 1)**	15.3	88.1	14	90	13.1	87.2
	(11.9-19.4)	(86.9-90.1)	(11.0-17.8)	(87.6-92.0)	(5.7-27.3)	(74.8-94.0)
**RaVL x duration**	8.9	98.2	11.8	97.5	7.8	97.8
	(6.3-12.4)	(97.2-98.9)	(9.0-15.3)	(96.1-98.4)	(2.7-20.8)	(88.8-99.6)

LVH: left ventricular hypertrophy. Note: Values of sensitivity and specificity are expressed with their respective 95% confidence intervals (95%CIs), using the McNemar's statistical test.

**Table 3:** DOR for ECG criteria for LVH according to age group

Criteria for LVH	GI (< 60 years)	GII (60-79 years)	GIII (≥ 80 years)
Perugia	5.2 (3.8-7.2)	4.2 (3.1-5.8)	6.8 (2.2-20.9)
(Rmax + Smax) product ≥ 2.8 mm.s	4.1 (3.0-5.5)	3.8 (2.8-5.2)	6.8 ( 2.0-23.0)
Peguero-Lo Presti	7.3 (4.6-11.3)	6.4 (3.9-10.4)	4.3 (1.3-13.7)
Narita	5.5 (4.0-7.4)	4.3 (3.2-5.8)	3.8 (1.09-13.4)
Romhilt-Estes	5.2 (3.3-8.1)	3.2 (2.1-5.0)	3.9 (0.9-15.9)
Cornell's voltage: ≥ 28 mm (h); ≥ 20 mm (m)	7.6 (4.7-12.3)	2.0 (1.4-2.8)	2.8 (0.7-10.3)
Sokolow-Lyon voltage ≥ 30 mm	3.6 (2.5-5.1)	3.3 (2.2-4.7)	3.9 (0.95-15.9)
Sokolow-Lyon voltage ≥ 35 mm	5.9 (3.6-9.7)	4.6 (2.7-8.0)	8.6 (0.99-75.12)
Cornell's voltage duration ≥ 2440 mm.ms	6.3 (4.1-9.7)	5.1 (3.3-7.8)	2.8 (0.7-10.3)
Gubner-Ungerleider ≥ 25 mV	8.1 (5.0-13.2)	6.2 (3.7-10.3)	2.7 (0.6-11.8)
RaVL ≥ 11 mm	3.8 (2.3-6.2)	3.2 (2.0-5.2)	1.9 (0.3-12.1)
V_6_/V_5_ratio > 1	1.3 (0.9-1.9)	1.4 (1.0-2.1)	1.03 (0.29-3.6)
RaVL.dur QRS > 103 mm.ms	5.5 (3.0-10.3)	5.4 (3.0-9.5)	3.9 (0.39-39.5)

Note: Data expressed as DOR and its respective 95% confidence interval (95%CI). HVE: left ventricular hypertrophy; DOR: diagnostic odds ratio.

In the assessment of reproducibility of ECG analysis, the level of interobserver agreement was 0.82 and 0.94 (kappa index), which are considered excellent numbers. The first figure corresponded to QRS duration, and the latter to the agreement of ECG criteria. ROC curves were constructed for the three groups studied with their respective areas under the curve (AUC) ( Figure 2 ).

**Figure 2:** ROC curve and area under the curve of the three studied grups.Group I (age <60 years); b) Group II (age 60-79 years); c) (age ≥80 years). OC: Receiver Operating Characteristic Curves, AUC: area under the curve.

## Discussion

LVH is an important cardiovascular risk factor, regardless of other manifestations or comorbidities. ^25^ Therefore, its detection by low-cost, easily available diagnostic methods is extremely relevant. In hypertensive patients, LVH is one of the most frequent pre-clinical manifestations of lesion of target-organ whose identification leads to changes in risk stratification and a more aggressive treatment. ^27^ Conversely, ECG is a low-cost test that has low Se but high Sp and reproducibility, which explains its wide use. However, this test may be influenced by several factors, such as obesity, smoking, gender and especially age. ^28^

The best assessment of ventricular mass is achieved by nuclear magnetic resonance imaging (NMRI); nevertheless, economic reasons make its routine use unfeasible in the evaluation of hypertensive patients. ^29^ In this sense, ECHO is used as a gold standard for the assessment of left ventricular mass with a high level of correlation and excellent intra- and interobserver reproducibility. In the present study, the reference test for the diagnosis of LVH was transthoracic ECHO. The modified Devereux formula was applied to calculate LV mass, which has a good correlation with the actual heart mass (r = 0.90; p < 0.001). ^30^

The older and very older population has been increasingly growing worldwide. It has already been acknowledged that the control of risk factors, which are highly prevalent in this population, increases their life expectancy. ^31^ Conversely, age is known to interfere with ECG Se in the detection of LVH. ^32^ With the purpose of identifying the best ECG criteria to diagnose the presence of LVH in older adults, an increasingly more frequent situation in doctor's offices and outpatient clinics, we assessed the main ECG indices described in the literature and used in epidemiological studies.

In our cohort, Perugia score was the criterion with the highest Se (44.7 %) in older and very older patients (35.6%) without leading to a significant loss in Sp. This criterion was described by Schillaci et al. ^16^ in 1994 and diagnoses LVH in hypertensive patients whose ECG findings show at least one of the three following parameters (strain pattern; modified Cornell's voltage criteria: SV_3_+ RaVL > 2.4mV in men and 2.0 mV in women; or Romhilt-Estes score ≥ 5). The authors reported Se of 34% and Sp of 93%, with a fair improvement in individual Se for the three criteria with no decrease in Sp. Although these authors investigate the performance of the proposed criteria with regard to gender and degree of LV mass, they did not mention the influence of age. In our study, patients younger than 60 years of age (Group I) had Se of 32.2% and Sp of 91.7%, percentages similar to those reported by Schillaci et al., ^16^ and there was a progressive increase in Se among older (Group II) and very older adults (Group III). ^16^

The criterion that considered the sum of the highest amplitude of the R wave and the highest S wave multiplied by QRS duration [(Rmax + Smax) x duration] also showed good Se in the very old population (39.4%), with Sp of 91.3%. In the original publication, there was no distinction between age groups, and Se and Sp were 35.2% and 88.7%, respectively. ^10^ Although being simple, this criterion had a result equivalent to that of Perugia score, because there was no statistically significant difference between the two criteria.

Recently, a new ECG criterion was proposed. The so-called Peguero-Lo Presti criterion had Se of 62% and Sp of 90%. ^17^ In our study, Se 34.2% and Sp of 89.3% in very older patients (Group III), and of 17.8% and 96.7% in older patients (Group II), respectively. Finally, 1270 patients younger than 60 years of age (Group I) showed Se 20.2% and Sp of 96.6%, results different from those reported here. We considered that the two samples were different; the sample assessed by Peguero- Lo Presti consisted of more severe patients with a high prevalence of LVH (60%). Obviously, diagnostic tests tend to have greater Se in a population of individuals with more severe disease.

In our sample, the percentage of LVH in the group of young, older, and very older adults were 44.7%, 36.0%, and 27.1%, respectively. The Narita criterion, which considers the sum of R wave in D_1_and the amplitude of S wave in V_4_, showed good Se in young and older adults (39.6% and 38.1%), respectively, but it reached only 26.3% in very older adults. Romhilt-Estes score, Cornell's voltage and duration, and Sokolow-Lyon ≥ 35 mm had very similar Se in the three age groups studied, with relatively low values ranging from 16.1 to 21%. Although recommended by several guidelines on arterial hypertension, these criteria had a lower performance. ^26^^,^^33^ The remaining assessed criteria in our cohort did not show satisfactory results with regard to Se, ranging from 8.9 to 18.5%.

The ECG indices that had the best performance took into account the amplitude of the S wave in V_3_or V_4_or the greatest S wave. This may be due to the fact that LVH generates higher vector projection of the QRS complex to the posterior horizontal plane. In LVH, the cavity grows posteriorly and to the left, changing the direction and the magnitude of the main depolarization vector. Hence, there will be an increase in the amplitude of the S wave in precordial V_3_and V_4_.

We found that most ECG criteria used in the diagnosis of LVH lose Se as sample age increases. However, this did not occur with regard to Perugia score and (Rmax + Smax) x duration criterion, especially in Group III. When we assessed the DOR, which evaluates the efficacy of a measure independently from prevalence and allows to estimate the overall efficacy of the parameter, we observed that the Perugia score and the (Rmax + Smax) x duration criterion yielded the highest values: DOR = 6.8. Thus, in Groups I and II, the greatest Se (39.6 and 38.1%) was observed for the Narita criterion, which also demonstrated high Sp (89.3% and 87.5%). However, for very old patients (Group III), the best performances for the diagnosis of LVH were found for Perugia score and the (Rmax + Smax) x duration, with Se of 44.7% and 39.4%; and Sp of 89.3% and 91.3%, respectively. The Sokolow-Lyon criteria, which have been widely used in several studies and may be the most well-known to physicians, due to the simplicity of its analysis, exhibited low Se in all age groups.

Our study showed that advanced age leads to loss of performance for several diagnostic criteria for LVH, exactly for a population at high cardiovascular risk. Therefore, the main contribution of our observations was detecting two ECG criteria that revealed to be superior in the detection of LVH among very older hypertensive patients. Furthermore, diagnostic imaging methods such as ECHO are not promptly available in many regions and health care facilities. Hence, ECG may be a useful, easily accessible and low-cost tool of practical interpretation and applicable to the diagnosis of LVH, using the criteria with better performance, especially in the very older population.

### Study limitations

In this study, coronary artery disease was ruled out by clinical history, specific imaging tests, or by the presence of pathological q waves on ECG. There was a lower number of patients in the group of very older adults compared to younger patients.

## Conclusions

The results obtained in this study suggest that, in very old adults with hypertension, the ECG criteria of Perugia and [(Rmax + Smax) x duration] showed the best diagnostic performance for the presence of LVH.
